# A New Particle Swarm Optimization-Based Method for Phase Unwrapping of MRI Data

**DOI:** 10.1155/2012/475745

**Published:** 2012-10-02

**Authors:** Wei He, Yiyuan Cheng, Ling Xia, Feng Liu

**Affiliations:** ^1^Department of Biomedical Engineering, Zhejiang University, Hangzhou 310027, China; ^2^School of Information Technology and Electrical Engineering, The University of Queensland, Brisbane, QLD 4072, Australia

## Abstract

A new method based on discrete particle swarm optimization (dPSO) algorithm is proposed to solve the branch-cut phase unwrapping problem of MRI data. In this method, the optimal order of matching the positive residues with the negative residues is first identified by the dPSO algorithm, then the branch cuts are placed to join each pair of the opposite polarity residues, and in the last step phases are unwrapped by flood-fill algorithm. The performance of the proposed algorithm was tested on both simulated phase image and MRI wrapped phase data sets. The results demonstrated that, compared with conventionally used branch-cut phase unwrapping algorithms, the dPSO algorithm is rather robust and effective.

## 1. Introduction

In magnetic resonance imaging (MRI), the complex signal contains both the magnitude and phase parts. Usually the magnitude of the MRI signal has been mainly considered. However, the phase of MRI signal offers very important information on the velocity of the moving spins, and can also be used to deduce useful information about the main *B*_0_ field inhomogeneity and the magnetic susceptibility variations [1]. In MRI, the phase information *ψ*_*i*,*j*_ is usually obtained from a complex MRI dataset *I*_*i*,*j*_ = |*I*_*i*,*j*_|exp⁡⁡(*ψ*_*i*,*j*_) through some mathematical operations, and the value always lies in the principal interval of (−*π*, *π*], consequently producing a wrapped phase *φ*_*i*,*j*_. This relationship can be described by *φ*_*i*,*j*_ = *W*(*ψ*_*i*,*j*_) = *ψ*_*i*,*j*_ ± 2*k*_*i*,*j*_ *π*, where *k*_*i*,*j*_ is an integer and *W* defines a wrapping operator that forces all values of its argument into the range (−*π*, *π*] by adding or subtracting an integral multiple of 2*π* radians from its argument. Phase unwrapping is the process of estimating the true phase *ψ*_*i*,*j*_ from the wrapped phase *φ*_*i*,*j*_. As an important tool, it can not only be used for the three-point Dixon water and fat separation, but also be applied to increase the dynamic range of phase contrast MR velocity measurements [2]. If the true phase gradients (i.e., the differences of *ψ*_*i*,*j*_) between contiguous pixels are less than **π** radians in magnitude in the entire space, the true phase can be unwrapped in a straightforward manner by just integrating the wrapped phase gradients [3]. However, the presence of the noise, undersampling, and/or object discontinuities often makes this condition unavailable. Therefore, the problem of phase unwrapping becomes complex in practice and difficult to solve, although significant amount of research effort has been devoted to date. In the literature, there are quite a few existing phase unwrapping algorithms [4], which can be grouped into two categories: path-following and minimum-norm methods [5].

**Figure 1:**
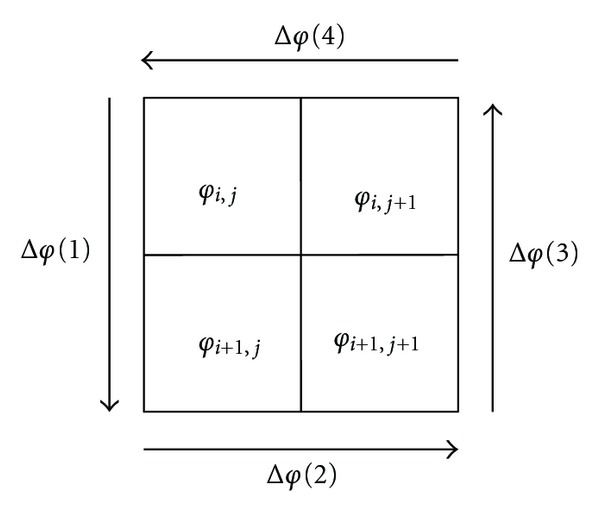
Residue calculation.

The branch-cut phase unwrapping method is one kind of the path-following methods. Unlike the minimum-norm methods, the branch-cut phase unwrapping technique offers correct phase unwrapping with no solution approximations [4]. In the operation, it locates residues, joins the residues by branch cuts, and then unwraps all the pixels avoiding those branch cuts. In the algorithm, residues are defined as local inconsistencies, which mark the starting and end of 2*π* discontinuities. Corresponding to formula (1), that is, the value *n* is 1 or −1 in a 2 × 2 closed-loop of the wrapped phase gradients [4], as shown in Figure 1:

(1) $$\begin{array}{c} \sum_{i=1}^{4}\Delta \varphi \left(i\right)=2\pi n. \end{array}$$

These wrapped phase gradients are computed counterclockwise:

(2) $$\begin{array}{c} \Delta \varphi \left(1\right)=W\left(\varphi_{i+1,j}-\varphi_{i,j}\right),\\ \Delta \varphi \left(2\right)=W\left(\varphi_{i+1,j+1}-\varphi_{i+1,j}\right),\\ \Delta \varphi \left(3\right)=W\left(\varphi_{i,j+1}-\varphi_{i+1,j+1}\right),\\ \Delta \varphi \left(4\right)=W\left(\varphi_{i,j}-\varphi_{i,j+1}\right). \end{array}$$

In formula (1), when *n* = 1, we label it a residue with a positive polarity. And when *n* = −1, the residue is labeled with a negative polarity. Otherwise it indicates there is no residue when *n* = 0. In this algorithm, the total length of branch cuts must been minimized, resulting a decrease of the amount of good pixels used as the branch cuts. This provides more unwrapping paths in dense residue areas, leading to a smoother result [5].

To achieve a minimum total length of branch cuts, various techniques have been developed for the branch-cut phase unwrapping method. These techniques include, for example, the Goldstein's algorithm [5, 6], the nearest-neighbor algorithm [7], the minimum-cost matching (MCM) algorithm [8], and the hybrid genetic algorithm (HGA) [4]. 

The nearest-neighbor algorithm is very efficient, but it utilizes local heuristics, thus causing some long branch cuts embedded in the phase image. Therefore, the distribution of the branch cuts does not achieve the optimum, and the resultant phase image is lack of the smoothness. The MCM is a graph theory-based algorithm, which uses the Hungarian algorithm to find the minimum total length of branch cuts. Although powerful, it is computationally expensive. The HGA employs a combination of global and local search methods, and its solution is usually good. However, the complexity of this algorithm tends to be a problem with the increase of residues.

Compared with these three methods, which place the branch cuts to connect pairs of residues of opposite polarity (called dipoles), the Goldstein's method joins the residues in clumps instead of pairs [5]. The Goldstein's algorithm is very efficient, but often forms some isolated patches.

In this paper, we propose a new discrete particle-swarm-optimization- (dPSO-) based branch-cut algorithm for phase unwrapping. The new dPSO algorithm is used to find the best way in which the negative polarity residues match with the positive ones, so that the overall length of the branch cuts is minimized. The performance of the new dPSO algorithm is compared with the Goldstein's and MCM algorithms.

## 2. Phase Unwrapping Using the Proposed Algorithm

### 2.1. Overview of the Basic PSO Algorithm

As an artificial intelligent algorithm, the particle swarm optimization (PSO) [9, 10], is easy for implementation (only few parameters to be adjusted) and converges fast.

In the PSO algorithm, the swarm consists of several particles, and each contains *N* elements. Then each particle is viewed as a point in an *N*-dimensional space. The *i*th particle of swam is represented as *U*_*i*_ = {*u*_*i*1_, *u*_*i*2_,…, *u*_*iN*_}. All the particles share their information and move to find the global optima. *P*_*i*_ = {*p*_*i*1_, *p*_*i*2_,…, *p*_*iN*_} represents that the local best position that the *i*th particle has reached, and *P*_*g*_ = {*p*_*g*1_, *p*_*g*2_,…, *p*_*gN*_} is the global best position. The velocity of the *i*th particle is *V*_*i*_ = {*v*_*i*1_, *v*_*i*2_,…, *v*_*iN*_}. Each particle of the swarm updates its velocity and position using the following formulas:

(3) Vit+1=w∗Vit+c1∗rand()∗(Pit−Uit)+c2∗Rand()∗(Pgt−Uit),

(4) $$\begin{array}{c} U_{i}^{t+1}=U_{i}^{t}+V_{i}^{t+1}, \end{array}$$

where *t* denotes the iteration number, *c*_1_ and *c*_2_ are learning factors (nonnegative constants), controlling (or regulating) the influence of *P*_*i*_ and *P*_*g*_, the function rand() and Rand() generate a random number ([0~1]), and *w* is the inertia weight factor.

A problem-specific fitness function (symbolized by *f*) is employed to measure the performance of each particle. Thereby, for a minimization problem, *P*_*i*_ and *P*_*g*_ can be found in current iteration as follows:

(5) $$\begin{array}{c} P_{i}^{t+1}=\{\begin{array}{cc} P_{i}^{t} & \text{if}\;\;f\left(P_{i}^{t}\right)\leq f\left(U_{i}^{t}\right)\\ U_{i}^{t} & \text{if}\;\;f\left(P_{i}^{t}\right)>f\left(U_{i}^{t}\right), \end{array} \end{array}$$

(6) $$\begin{array}{c} P_{g}^{t}=\arg\underset{P_{i}^{t}}{\min}\left[f\left(P_{i}^{t}\right)\right]. \end{array}$$

To date the PSO technique has been well developed for the continuous problem, but not in discrete domain.

### 2.2. The dPSO Algorithm for Phase Unwrapping

#### 2.2.1. Particle and Swarm Initialization

Any problem adopting the PSO algorithm has to be interpreted into PSO particle form. For phase unwrapping, every particle should be composed of some elements corresponding to the indexes of residues. If we calculate all the residues in the wrapped phase image at one stroke, sometimes the size of the particle may be too large and the swarm size required may be enlarged accordingly. This may result in a poor solution and/or extending the convergence time. To avoid this, the whole image is divided into sub regions and therefore the residues are set into different small groups.

The process can be described as follows.

(1) The image is partitioned on the basis of its phase derivative variance map. The phase derivative variance is defined as follows [5]:
  (7) $$\begin{array}{c} Z_{m,n}=\frac{\sqrt{\sum {\left(\Delta \varphi_{i,j}^{x}-\overset{-}{\Delta \varphi_{m,n}^{x}}\right)}^{2}}+\sqrt{\sum {\left(\Delta \varphi_{i,j}^{y}-\overset{-}{\Delta \varphi_{m,n}^{y}}\right)}^{2}}}{l^{2}}, \end{array}$$
  where for each sum the indexes (*i*, *j*) cover over the *l* × *l* window centered at the pixel (*m*, *n*). The terms Δ*φ*_*i*,*j*_^*x*^ and Δ*φ*_*i*,*j*_^*y*^ are the wrapped phase gradients in the *l* × *l* windows, and $\overset{-}{\Delta \varphi_{m,n}^{x}}$ and $\overset{-}{\Delta \varphi_{m,n}^{y}}$ are the averages of these wrapped phase gradients. In this paper *l* = 3.
(2) Based on an appropriate threshold, the phase derivative variance map can be converted into a binary one, where the low phase derivative variance values turns out to be 0 and the high ones becomes to be 1. In this way the image is divided into separate areas. To obtain a proper threshold, a classical approach called Otsu's method [11] has been adopted in this study.
(3) It is observed that the majority of residues cluster in the patches of value 1. Thus the residues are grouped by taking the above steps.

In each region the indexes of the residues are inserted in two arrays regardless of the order. One is a positive polarity residue array, and the other is a negative polarity residue array. Provided there are *M* positive polarity residues and *N* negative polarity residues in one region, respectively, the positive residue array and the negative one are accordingly denoted as {*s*_1_, *s*_2_,…, *s*_*M*_} and *U*_*i*_ = {*u*_*i*1_, *u*_*i*2_,…, *u*_*iN*_}. The former will be fixed throughout all generations and serves as a reference. And the later acts as a particle *U*_1_ of the initial swarm. The rest particles of the swarm are generated via arranging the order of the elements in *U*_1_ in a random manner.

#### 2.2.2. Fitness Estimate

From the abovementioned, it can be easily seen that the dPSO algorithm is used to find out the best matched order of the elements in the particle with the reference array.

In dPSO, the quality of the current solution is judged by the fitness function. Since the branch-cut phase unwrapping must minimize the total length of branch cuts, the corresponding fitness function is obviously for calculating the total length of branch cuts in the wrapped phase image. Here we employ Euclidian distance to assess the total cut length:

(8) $$\begin{array}{c} f=\sum_{j}^{\min(M,N)}\sqrt{{\left(x_{s_{j}}-x_{u_{ij}}\right)}^{2}+{\left(y_{s_{j}}-y_{u_{ij}}\right)}^{2}}, \end{array}$$

where *x* and *y* denote the residue's *x*-coordinate and *y*-coordinate.

#### 2.2.3. Velocity Update

Owing to the attribute of dPSO algorithm for branch-cut phase unwrapping, the iterative velocity in formula (3) should be a set of permutation operators rather than a usual vector. Various permutation operators have been introduced for discrete particle swarm optimization. Here we choose the adjustment operator [12], not the swap operator [13, 14], as the permutation operator. This is because compared with the swap operator, the adjustment operator avoids returning to the previous position [12]. 

 In addition, during all the iterations, *w* is set to be linearly varied ([0.9~0.4]) [10]. This setting considers the global searching capacity and convergence rate of the optimization:

(9) $$\begin{array}{c} w=0.9-0.5\times \left(\frac{t}{T}\right), \end{array}$$

where *T* is the maximal iteration times.

To make formula (3) suitable for the dPSO operation, some concepts are given as follows.

**Definition 1**

The adjustment operator AO(*k*, *l*) is defined as deleting the element in the *k*th position and popping it in the *l*th position in the array.

For example, AO(5,3) acting on the array *U*_*i*_ = {5,1, 4,2, 3} gets a result *U*_*i*_′ = {5,1, 3,4, 2}.

**Definition 2**

One or more adjustment operators make up an adjustment sequence (AS). That is AS = {AO(*k*_1_, *l*_1_), AO(*k*_2_, *l*_2_),…, AO(*k*_*n*_, *l*_*n*_)}.

Acting an AS on an array means that every adjustment operator of the sequence acts on the array in turn. Therefore, in the AS, it is critical to properly arrange the order of adjustment operators.

**Definition 3**

The plus sign “+” between ASs has its new meaning. It is defined as forming a new longer AS by putting the latter behind the former.

For example, AO(2,4) + AO(5,4) = {AO(2,4), AO(5,4)},  {AO(2,4), AO(5,4)} + AO(3,1) = {AO(2,4), AO(5,4), AO(3,1)},  {AO(2,4), AO(5,4)}+{AO(1,3), AO(5,4)} = {AO(2,4), AO(5, 4), AO(1,3), AO(5,4)}. This operation does not satisfy the commutative law.

**Definition 4**

The minus sign “−” between two arrays means constructing an AS which can act on the array after minus sign to obtain the array before.

Supposing there are two arrays, according to the array before minus sign from left to right, this AS can be obtained by adjusting the order of the array after. For example, in *W* − *R*, *W* = {1,4, 3,2, 5} and *R* = {1,5, 4,2, 3}. *W*(2) = *R*(3), so the first adjustment operator of AS is AO(3,2), and the first new array *R*′ = {1,4, 5,2, 3}. Then *W*(3) = *R*′(5), so the second adjustment operator is AO(5,3), and the second new array is *R*′′ = {1,4, 3,5, 2}. *W*(4) = *R*′′(5), so the third adjustment operator is AO(5,4), and we finally obtain the same array as *W*. Thus the calculation ends and AS = *W* − *R* = {AO(3,2),  AO(5,3),  AO(5,4)}.

**Definition 5**

The multiplication sign “∗” between two real numbers is simply the operation of multiplication.

**Definition 6**

The sign “∗” between a real number and an AS means reserving a certain amount of the adjustment operators in the AS in turn when the real number is in the range (0, 1).

Given that the number of adjustment operators in the AS denotes as ||AS||, the number of retaining adjustment operators is [*b*||AS||], which rounds the product of *b* and ||AS|| to the nearest integer.

#### 2.2.4. Position Update

Similarly, the formula (4) has to be redeclared to fit the requirement of the dPSO.

**Definition 7**

The plus sign “+” between an array and an AS means acting the adjustment operators of the AS on the array in order. For example, given *U*_*i*_ = {5,1, 3, 2,4} and AS = {AO(2,1),  AO(2,5)}, *U*_*i*_ + AS = {1,3, 2,4, 5}.

It is obvious that the operations defined in Definitions 4 and 7 are inverse to each other.

#### 2.2.5. The Procedure Description of dPSO Algorithm for Phase Unwrapping

The process of using the dPSO algorithm for phase unwrapping is summarized as follows.

1. According to Section 2.2.1, the residues in the image are divided into several clusters. Set *h* = 1.
2. Do the following steps in the *h*th group of residues.
3. Set values to the parameters of dPSO, which include the learning factors (*c*_1_, *c*_2_), the maximal iteration times (*T*), and the number of particles in swarm, and also the termination criteria.
4. Initialize the swarm according to Section 2.2.1. Each particle has its random velocity, that is, AS. Set *t* = 1.
5. Evaluate the fitness of every particle according to formula (8) and find the current *P*_*i*_ *P*_*g*_ by formula (5), (6), respectively. Calculate the current inertia weight factor according to formula (9).
6. Set *t* = *t* + 1. Use formula (3) to get the new velocity *V*_*i*_. Then calculate the new position *U*_*i*_ according to formula (4).
7. Repeat (5)-(6) until *t* = *T* or meeting the termination criteria.
8. *P*_*g*_ is the best indexes order of the negative polarity residues matched with the positive ones in this group.
9. Set *h* = *h* + 1. Repeat ([Disp-formula EEq2])–(8) until *h* equals the number of the residues groups plus 1.

### 2.3. Branch Cuts and Unwrapping

Once the best match in every group has been found by dPSO, each pair of two matching opposite polarity residues are connected by the branch cuts. It is worth mentioning that, owing to that the number of opposite polarity residues is not always equal to each other, there are usually one or more residues left in each group. Then the nearest-neighbor algorithm [7] is employed to place branch cuts to balance these remaining residues. So far all the residues have been balanced by branch cuts.

Finally, the phase data can be unwrapped by flood-fill algorithm [15, 16], without crossing the branch cuts as follows:

1. Choose a start pixel, whose phase value is stored as an unwrapped phase value in the solution matrix. The four neighboring pixels are unwrapped next and their unwrapped phase values are placed in the solution matrix. These four pixels are inserted in the unwrapped list.
2. Pick (and then eliminate) a pixel from the unwrapped list. Unwrap the phase values of its four neighboring pixels, avoiding pixels that have been unwrapped. Insert these pixels in the unwrapped list and put their unwrapped phase values in the solution matrix.
3. Repeat (2) until the unwrapped list becomes empty.

In fact, it does not always mean that all the pixels have been unwrapped when the unwrapped list becomes empty. Because sometimes there are some pixels in the image encircled by the branch cuts, they cannot be unwrapped if not crossing the branch cuts.

### 2.4. Weighted *L* ^0^ Measure

Weighted *L* ^0^ measure, the most general/practical error measure to consider [5], is used to evaluate the quality of an unwrapped solution:

(10) ε=1rc[∑i=1r−1∑j=1cwi,jx||ψ(i+1,j)−ψ(i,j)−Δφi,jx||0  +∑i=1r∑j=1c−1wi,jy||ψ(i,j+1)−ψ(i,j)−Δφi,jy||0],

where *r* and *c* are the number of rows and columns, *w*_*i*,*j*_^*x*^ and *w*_*i*,*j*_^*y*^ are user-defined weights, and the *L* ^0^ norm measures a count of the number of pixels at which the gradients of the unwrapped solution mismatch the wrapped phase gradients. In this paper the weights adopted are derived from the quality map mentioned above, not just omitted (i.e., equal to 1).

## 3. Results and Discussion

We have tested the performance of the proposed algorithm on both simulated and MRI phase data on a PC (Intel 2 Quad CPU 2.39 GHz, MATLAB). We set both learning factors (*c*_1_, *c*_2_) used in dPSO to be 2. The results were compared with the well-known Goldstein's and the MCM algorithms.

### 3.1. Simulation Results

**Figure 2:**
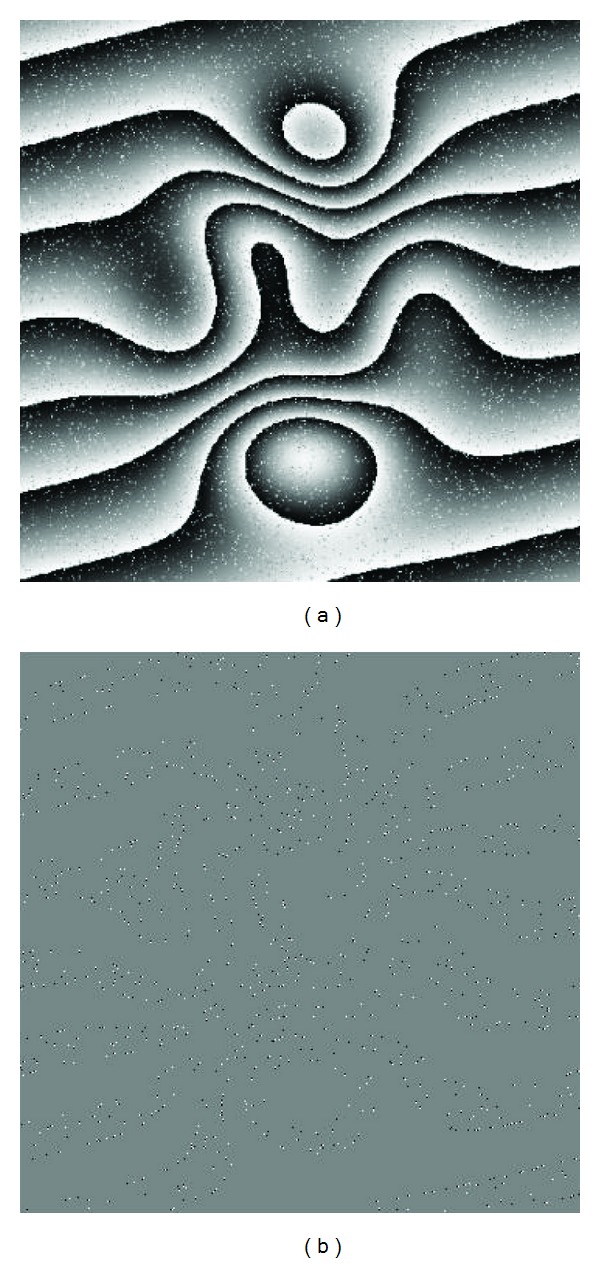
(a) A 512 × 512 simulated wrapped phase image, (b) its residue distribution including 2460 residues, 1231 positive polarity residues (white pixels), and 1229 negative polarity residues (black pixels).

The proposed algorithm was implemented on a simulated wrapped phase image with salt and pepper noise (the signal-to-noise ratio is 7.58 dB), which has 2460 residues.  Figure 2(a) shows the simulated wrapped phase image. And its residue distribution is shown in Figure 2(b), where the positive and negative residues are marked as white and black pixels, respectively.

**Figure 3:**
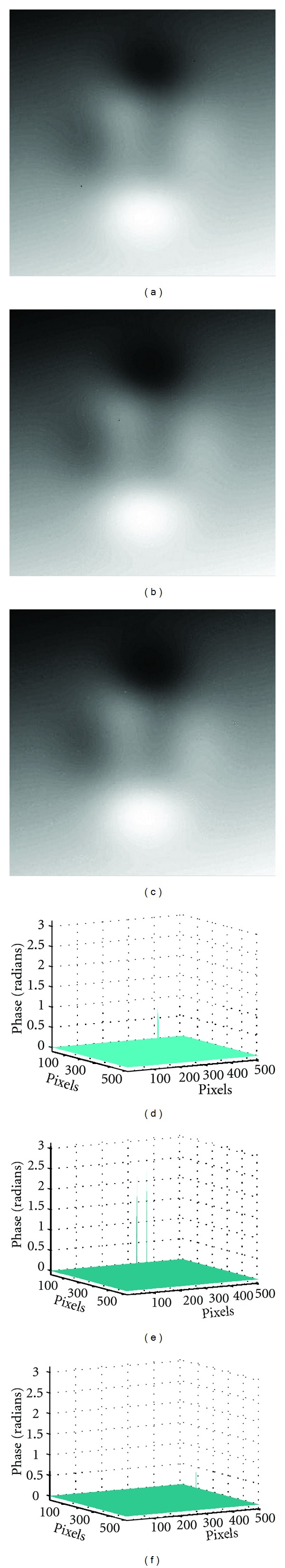
The top row is the unwrapped phase image for the simulated wrapped phase map in Figure 2(a) achieved by (a) dPSO, (b) Goldstein's, and (c) MCM algorithms. The bottom is the corresponding difference map got by (d) dPSO, (e) Goldstein's, and (f) MCM algorithms.

The resultant unwrapped phase image in Figure 3(a) was achieved by dPSO using a swarm of 300 particles and *T* = 1000. Figures 3(b) and 3(c) depict the corresponding unwrapped phase images obtained by Goldstein's and MCM algorithms, respectively. We rewrapped these resultant solutions and subtracted the original wrapped phase data from them. The corresponding difference maps, which are plotted in 3D visualization, are shown in Figures 3(d)–3(f). As mentioned in Section 1, the difference between the wrapped phase and the true phase is an integer multiples of 2*π*. As a result, these curve surfaces of difference maps give a visual representation of the deviations between the true phases and the results, which can illustrate the accuracy of the unwrapped solutions directly. The average value of the difference map is called *average difference*.

The performance of dPSO with respect to the other two algorithms can clearly be seen in Table 1. In terms of weighted *L* ^0^ measure, average difference, and total cuts length, the dPSO algorithm is better than the Goldstein's, but not as good as the MCM. On the other hand, the execution time of dPSO is much less than that of the MCM, but not comparable to that of the Goldstein's.

### 3.2. Results of MRI Data

**Figure 4:**
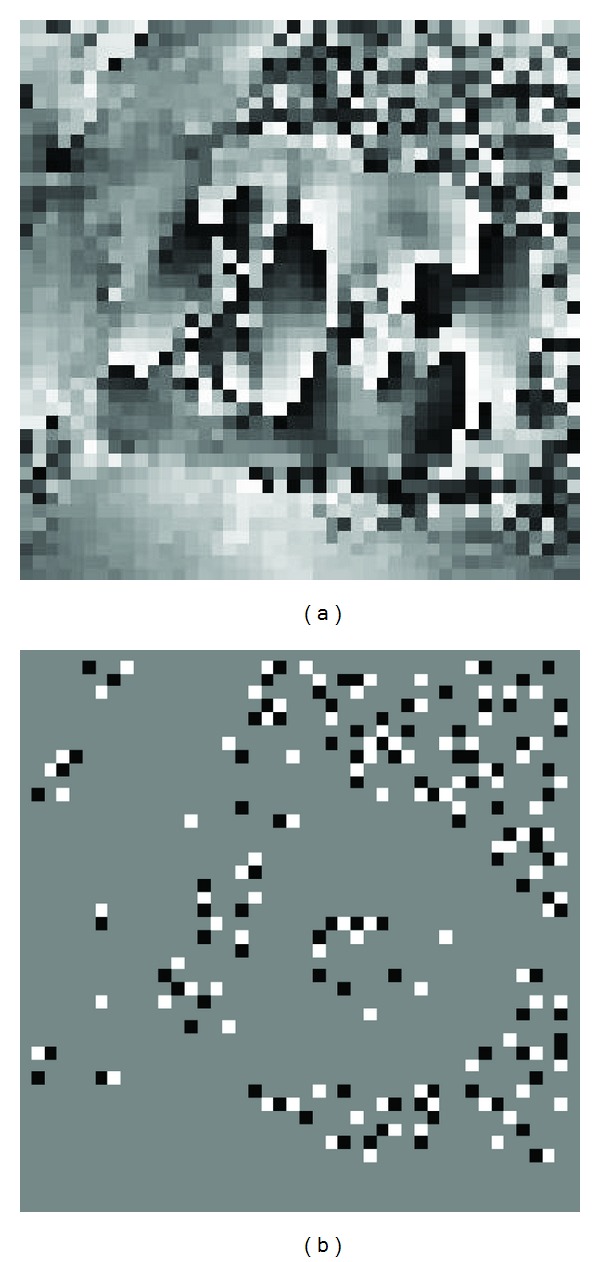
(a) A 44 × 44 displacement encoded MRI heart phase image, (b) its corresponding residue distribution involving 396 residues, 198 positive polarity residues, and 198 negative polarity residues.

The proposed algorithm was also executed on a displacement encoded MRI heart phase data set [17] with 396 residues. The wrapped phase image and its corresponding residue distribution are shown in Figure 4.

**Figure 5:**
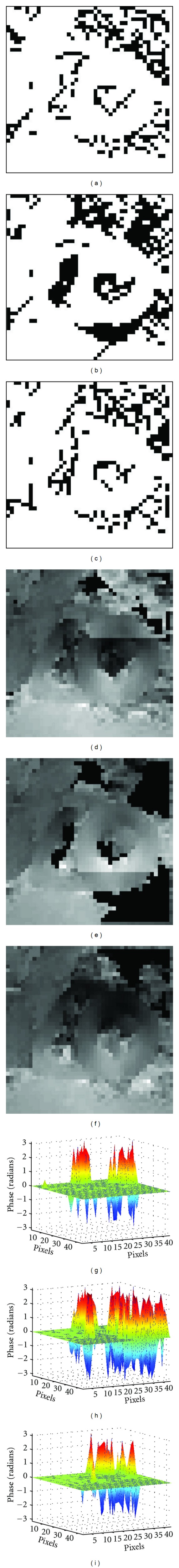
The top row is the branch-cut distribution for the MRI heart phase image in Figure 4(a) achieved using (a) dPSO, (b) Goldstein's, and (c) MCM algorithms. The middle is the corresponding unwrapped phase result of (d) dPSO, (e) Goldstein's, and (f) MCM algorithms. The bottom is the corresponding difference map of (g) dPSO, (h) Goldstein's, and (i) MCM algorithms.

Figures 5(a)–5(c) depict the branch-cut distribution achieved by the three algorithms, where the black pixels mark the branch cuts. The dPSO result in Figure 5(d) was obtained by using a swarm of 300 particles. And the results of the other algorithms are shown in Figures 5(e) and 5(f), respectively. In Figure 5(e) several patches are isolated, two large ones in the upper part, two small ones in the middle, and a very large one in the lower right part. Compared with Figures 4(b) and 5(b), it is easy to observe that these patches are completely isolated by branch cuts, which would lead to an incorrect unwrapping. In addition, the isolated areas tend to arise in the regions with dense residues, because in such regions the branch cuts are often close to each other and have more possibility to encircle some pixels. However, in Figures 5(d) and 5(f) the isolated patches are much smaller and less. The dipole branch-cut methods appear to be less likely to isolate regions in the phase image by branch cuts, since the branch cuts balance the residues in pairs not in clumps. The difference maps of the three approaches are then generated like in Section 3.1, shown in Figures 5(g)–5(i). Intuitively, both the dPSO and MCM produce more desirable results than the Goldstein's. 

As shown in Table 2, the Goldstein's algorithm is extremely fast. Neither dPSO nor MCM is comparable to it. But the proposed approach has the smallest weighted *L* ^0^ measure and average difference. In addition, its total cuts length is the shortest.

**Figure 6:**
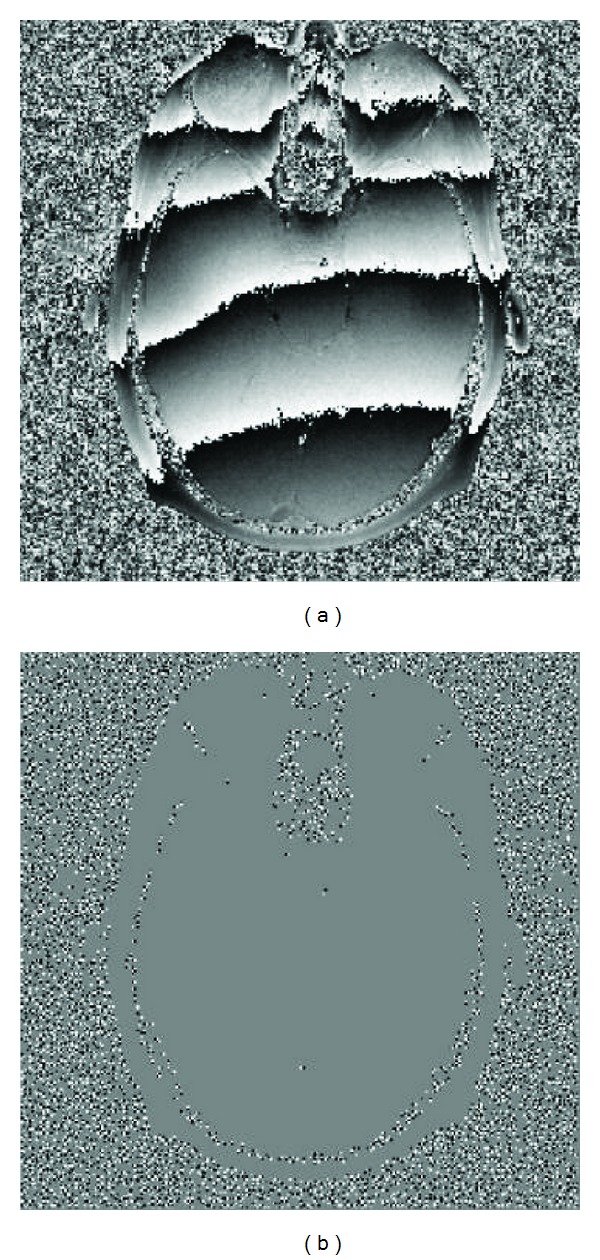
(a) A 256 × 256 MRI head phase image, (b) its corresponding residue distribution containing 9795 residues, 4904 positive polarity residues, and 4891 negative polarity residues.

**Figure 7:**
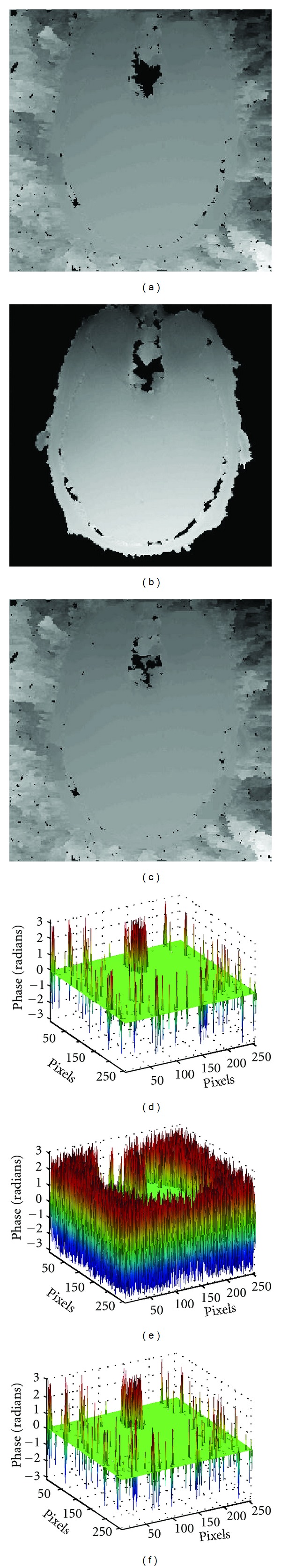
The top row is the unwrapped phase image for the MRI head phase image in Figure 6(a) achieved by (a) dPSO, (b) Goldstein's, and (c) MCM algorithms. The bottom is the corresponding difference map got by (d) dPSO, (e) Goldstein's, and (f) MCM algorithms.

Another example is the MRI head phase data. The wrapped phase image is shown in Figure 6(a).  Figure 6(b) depicts its residues distribution. The unwrapped phase images achieved by the dPSO, Goldstein's, and MCM algorithms are displayed in Figures 7(a)–7(c), respectively. Obviously, in Figure 7(b) the surrounding area was not unwrapped at all. According to the previous analysis, the Goldstein's method isolates this area by branch cuts. In comparison to Figure 6(a), the surrounding part roughly is the back ground with dense noise. However, due to the property of dipole branch-cut phase unwrapping method the branch cuts could hardly enclose the surrounding area, which certainly caused unwrapping phases in this area. The same inferences can be also made according to the difference maps of three methods shown in Figures 7(d)–7(f).

The weighted *L* ^0^ measure, average difference and total cuts length are calculated over the whole image whether the pixel is inside the region of interest (ROI) or background. Thereby in these three respects, as shown in Table 3, the dPSO and MCM algorithms are much better than the Goldstein's. Though the dPSO method does not get a better solution than that of the MCM, there is little difference between them. That is, dPSO is comparable to MCM. Furthermore the former converges nearly 68% faster than the latter.

Viewing dPSO's performance on these three examples, we can find that the dPSO algorithm takes more time to achieve an optimal solution when plenty of residues uniformly scatter throughout large areas. This is because the pixels in each of these areas often have similar quality and then the residues in each area can hardly be separated into more than one group, which results in the increase of the particle size for every group.

## 4. Conclusions

We have presented a new branch-cut phase unwrapping method based on dPSO algorithm in this paper. Both simulated and real wrapped phase data were used to test the performance of the proposed algorithm. The results of dPSO were compared with the Goldstein's and the MCM algorithms. It was found that the dPSO method is better than Goldstein's algorithm in terms of weighted *L* ^0^ measure, average difference and total branch cuts length. Moreover, the dPSO is much faster than the MCM algorithm in getting a global optimum solution while it is comparable to the latter in terms of weighted *L* ^0^ measure, average difference and total branch cuts length. Generally speaking, it has been demonstrated to be robust, effective for the phase unwrapping application.

In addition, it is capable of dealing with large branch-cut problem with thousands of residues. The complexity of the dPSO algorithm increases when the number of residues in a group increases, as the length of the particle extends which requires a larger swarm size. Future research will make this algorithm to be more efficiently operated for the phase unwrapping study.

## Figures and Tables

**Table 1:** Comparing dPSO with other algorithms for the simulated phase image in Figure 2(a) in terms of weighted *L* ^0^ measure, average difference, total cuts length, and execution time.

Algorithm	Weighted *L* ^0^ measure	Average difference (radian)	Total cuts length	Execution time (s)
dPSO	0.001301	0.873*e* − 5	1310	1328
Goldstein's	0.001384	3.77*e* − 5	1313	79
MCM	0.001286	0.365*e* − 5	1298	3513

**Table 2:** Comparing dPSO with other algorithms for the displacement encoded MRI heart phase map in Figure 4(a) in terms of weighted *L* ^0^ measure, average difference, total cuts length, and execution time.

Algorithm	Weighted *L* ^0^ measure	Average difference (radian)	Total cuts length	Execution time (s)
dPSO	0.052223	0.056251	310	142
Goldstein's	0.109667	0.294767	468	8
MCM	0.052490	0.076967	317	203

**Table 3:** Comparing dPSO with other algorithms for the MRI head phase map in Figure 6(a) in terms of weighted *L* ^0^ measure, average difference, total cuts length, and execution time

Algorithm	Weighted *L* ^0^ measure	Average difference (radian)	Total cuts length	Execution time (s)
dPSO	0.064380	0.025642	9871	979
Goldstein's	0.156827	0.665523	25533	41
MCM	0.063179	0.021957	9846	3052
